# Factors associated with sleep disorders among university students in Jiangsu Province: a cross-sectional study

**DOI:** 10.3389/fpsyt.2024.1288498

**Published:** 2024-02-23

**Authors:** Bin Hu, Qi Wu, Yue Wang, Haitao Zhou, Dehui Yin

**Affiliations:** Key Laboratory of Human Genetics and Environmental Medicine, School of Public Health, Xuzhou Medical University, Xuzhou, China

**Keywords:** university students, sleep quality, psychological resilience, PSQI, MPAI, influencing factors

## Abstract

**Objective:**

This study aims to establish the precise prevalence of sleep disorders among university students in Jiangsu Province. Utilizing a representative sample of students, we measured their sleep quality based on the Pittsburgh Sleep Quality Index (PSQI). Our objective is to quantitatively assess the magnitude of sleep quality and identify key factors. By detailed analysis of these relationships, our study seeks to provide actionable insights for the development of targeted interventions to enhance sleep quality within this population.

**Methods:**

From October to November 2022, we conducted a cross-sectional web-based survey in Jiangsu Province, China. Using convenient cluster sampling in each college, a total of 8457 participants were selected. The PSQI was applied to assess sleep quality among university students. Data collected included sociodemographic details, scores from the Mobile Phone Dependence Index (MPAI) and psychological resilience measured by the Connor-Davidson Resilience Scale (CD-RISC).

**Results:**

The overall prevalence of poor sleep quality among the participants was 39.30%. Binary logistic regression analysis revealed that higher physical activity (OR = 0.921; 95% CI: 0.779-1.090), earlier roommate bedtimes (OR = 0.799; 95% CI: 0.718-0.888), quieter dormitories (OR = 0.732; 95% CI: 0.647-0.828) and higher psychological resilience (OR = 0.982; 95% CI, 0.979-0.984) were protective factors linked to lower risk of poor sleep quality. Conversely, being a female student (OR = 1.238; 95% CI: 1.109-1.382), being a senior (OR = 1.582; 95% CI: 1.344-1.863), single-child status (OR = 1.195; 95% CI: 1.077-1.326), regular smoking (OR = 1.833; 95% CI: 1.181-2.847), regular alcohol consumption (OR = 1.737; 95% CI: 1.065-2.833), high academic stress (OR = 1.326; 95% CI: 1.012-1.736), high employment stress (OR = 1.352; 95% CI: 1.156-1.582), dissatisfaction with dormitory hygiene (OR = 1.140; 95% CI: 1.028-1.265), poor self-rated physical health (OR = 1.969; 95% CI: 1.533-2.529), poor self-rated mental health (OR = 2.924; 95% CI: 2.309-3.702) and higher mobile phone dependency were risk factors associated with an increased likelihood of poor sleep quality.

**Conclusion:**

The sleep quality among university students should attract immediate attention. The development of public services and mental health education initiatives is crucial in enhancing the sleep health of this population.

## 1 Introduction

Early studies highlighted the importance of sleep duration for health (1–3), whereas recent research has pivoted attention towards sleep quality. University students normally face poor sleep quality (4) due to social influences, health conditions and academic workload. Sleep deprivation in university students can greatly impinge on students’ cognitive function and scholastic achievements (5, 6), and is also linked to serious health problems like obesity (7) and mental issues including depression, anxiety (8, 9), stress, and burnout (10, 11). Researchers have applied the Pittsburgh Sleep Quality Index across various cultural backdrops to delineate between good and poor sleepers among students (12–14). A spectrum of determinants, from sociodemographic to lifestyle and mental health factors, have been scrutinized for their impact on sleep. Gender appears to play an ambiguous role, with some studies noting that female students tend to have lower quality sleep (15, 16), while others found that there was no significant difference between gender (17, 18). However, influence of Body Mass Index (BMI) were more consistent with higher BMI associated with increased sleep issues (2, 19). Academic advancement has also been identified as a predictor of sleep disturbances, as students in higher grades have lower sleep quality (18, 20). Poor accommodations have also been found to affect sleep comfort and quality (21, 22). A study revealed that family income or wealth can serve as supportive resources for promoting healthy sleep among university students (23), and evidence from cross-sectional studies suggests that students from lower socioeconomic backgrounds are more likely to report sleep disturbances and poor sleep quality (24). Additionally, a study found that poor socioeconomic status increases the risk of developing sleep disorders (25). Risky behaviors such as lack of physical activity (26), smoking (27) and regular alcohol consumption (17) are common among university students and contribute to poor sleep quality in this population. Academic stress has also been identified as a significant factor associated with poor sleep quality among university students. Academic stress exacerbates sleep challenges, often causing students to sleep late (28). Taken together, entering university can be considered a challenging stage in the lives of adolescents, and high levels of perceived stress have been identified as a central predictor of poor sleep quality among university students (29).

Smartphone addiction refers to online behavioral dependence where excessive use of the internet, video games and mobile devices leads to unconscious exposure to the detrimental effects of technology (30). Numerous reports have shown that excessive smartphone use can result in sleep disorders (31, 32). Encouraging physical activity has been suggested as a counteractive measure to this problem (33). Therefore, it is anticipated that the addictive nature of mobile phones may serve as a positive predictor of sleep disorders.

Psychological resilience has emerged as a potential shield against sleep impairment. Studies revealed an inverse relation between resilience levels and the occurrence of sleep disorders (34, 35). Therefore, psychological resilience can serve as a crucial protective factor against sleep disorders.

In summary, lots of studies has been dedicated to explore the factors affecting sleep quality, and many of these studies have approached the issue from a singular perspective or with a narrow focus. Present research has identified various elements, such as sociodemographic, lifestyle, mental health and behavioral patterns, which can influence sleep quality. However, there is a notable gap in a comprehensive approach that integrates these diverse determinants to offer a more holistic understanding of sleep quality among university students. Therefore, a study that amalgamates these factors, specifically tailored to the context of university students in Jiangsu Province, China, is necessary and timely.

## 2 Data collection procedures

### 2.1 Study design and period

Between October 27 and November 27, 2022, an online questionnaire was distributed to university students across Jiangsu Province using a convenient cluster sampling method. The survey involved students from 34 higher education institutions, including public and private universities with various sizes and specializations, which can offer a broad perspective of the educational landscape within this economically varied region. A web-based platform aims at protecting participants’ secret, the survey obtained informed consent electronically, students were aware of the study’s objectives, the anonymous nature of their responses and their right. The choice of data collection period during the academic semester was deliberated to optimize student engagement and response rates.

### 2.2 Inclusion and exclusion criteria

During the data collection period, all university students enrolled in Jiangsu Province were included in the study, excluding individuals who were unable to provide accurate data as required by the study, such as those who lacked data records or cooperation.

### 2.3 Sample size determination

Estimated according to the sample size calculation formula:


$$n=z_{\alpha }^{2}\times pq/d^{2}$$

**Figure 1:**
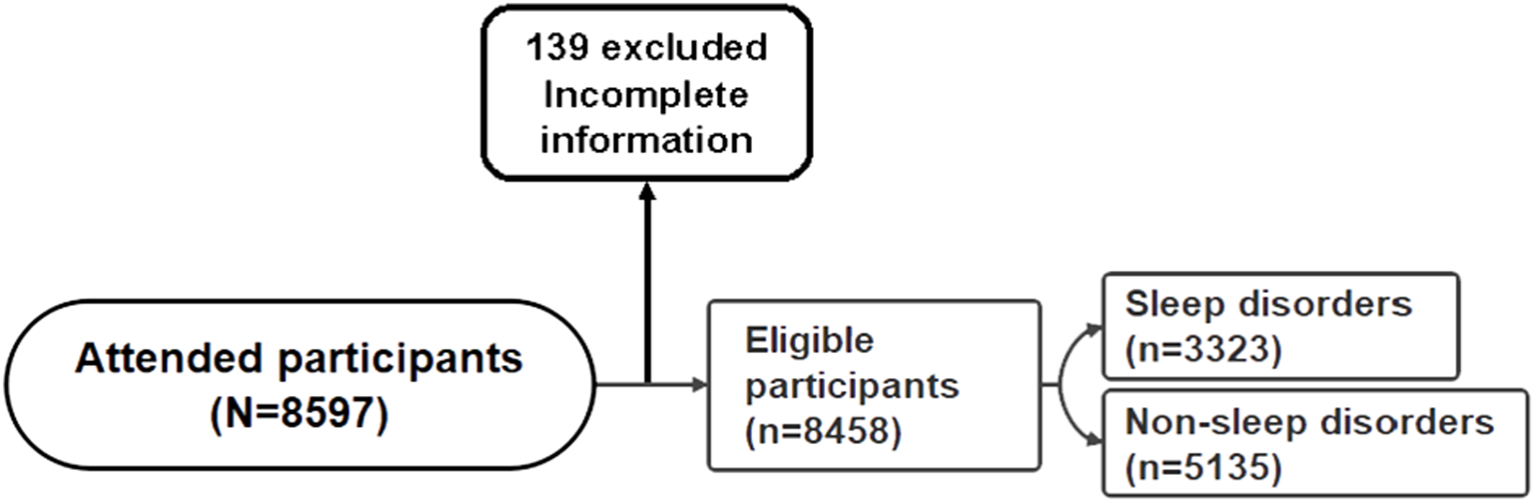
Flowchart of study participants.

When α =0.05, $z_{\alpha }=1.96$ . Let p be the expected prevalence rate, and q=1−p . Based on the preliminary survey results of this study, the estimated detection rate of sleep disorders among college students is p=16% . The allowable error is d=0.1×p , and the significance level is α =0.05. As a result, the estimated sample size for the survey is 2017. Initially, the study included 8597 participants, excluding 139 individuals providing incomplete information (**Figure 1**). Subsequently, the final analysis included 8458 participants, the validity rate of the questionnaire was 98.4%.

## 3 Data collection and tools

### 3.1 Demographic data

This study assessed an array of demographic characteristics: gender (categorized as male or female), grade level (broken down by First-year, Second-year, and Third-year or higher), native place (classified as Rural or Urban), status as an only child (designated as Only child or Not only child), parental education level (divided into High school and Below, Junior university, or Undergraduate and above), family’s economic status (identified as High, Middle, or Low), BMI (segmented into <18.5, 18.5-24, and >24), smoking and drinking habits (Never, Occasional, or Usually), frequency of physical activity (categorized as ≤1 per month, 1-3 times per week, or 4-7 times per week), levels of academic and employment-related pressure (considered Low, Moderate, or High), occurrence of roommates sleeping late (Yes or No), presence of noise in the dormitory (Yes or No), intensity of dormitory lighting at night (Yes or No), acknowledgment of dormitory cleanliness (Yes or No), quality of relationships with classmates (rated as Good, General, or Poor), self-evaluated physical and mental health status (rated as Good, General, or Poor).

### 3.2 Pittsburgh sleep quality index

The PSQI serves as an tool to evaluate sleep quality (36). This self-administered questionnaire comprises of 18 items, 16 of which are scored individually, omitting data on bedtime and wake-up time - and is engineered to gauge an individual’s subjective assessment of their sleep patterns over one month. Each item score ranges from 0 to 3, and the aggregated total yields the overall sleep quality score, which spans from 0 to 21, wherein higher totals signify diminished sleep quality. A global PSQI score > 5 indicates poor sleep quality (36). The PSQI has been proven to be a reliable and effective tool for assessing the sleep quality of the Chinese population (37). In this study, the Cronbach’s alpha for the PSQI was 0.877 and the Kaiser-Meyer-Olkin value was 0.925.

### 3.3 Mobile phone addiction index

The mobile phone addiction index (MPAI) revised by Leung (38) is widely recognized for its efficacy in evaluating mobile phone addiction, particularly among university students. The scale comprises 17 items, each scored on a scale of 1 to 5, the higher total score, the greater the indication of addiction to mobile phones. The full scale can be divided into four dimensions: “Inability to control craving,” “Anxiety and Feeling Lost,” “Withdrawal and escape,” and “Productivity loss.” In this study, the Cronbach’s alpha for the MPAI was 0.925 and the Kaiser-Meyer-Olkin value was 0.917.

### 3.4 Connor-Davidson resilience scale

The Connor-Davidson Resilience Scale (CD-RISC) (39) is a tool to measure the ability to cope with stress and adversity. This study used a revised version (40) translated by Nan Xiao to measure the level of psychological resilience in university students. This scale contains 25 question entries which were divided into three dimensions of “Optimism”, “Self-improvement” and “Tenacity”, using a 5-point Likert scale: not true at all (0) to true almost all the time (4). The total score ranges from 0 to 100, with a higher score indicating more resilience. In this study, the Cronbach’s alpha for the CD-RISC was 0.969 and the Kaiser-Meyer-Olkin value was 0.979.

## 4 Statistical analysis

Statistical assessment was conducted utilizing SPSS software, version 26.0. Missing data were addressed through methods such as listwise deletion and mean imputation. The threshold for determining statistical significance was established at a P-value less than 0.05. To characterize the study’s sample, descriptive statistics were computed, including means, standard deviations for continuous variables, frequencies and percentages for categorical variables. Differences among categorical demographic variables, mobile phone addiction and sleep quality were examined using chi-square tests. For continuous variables, t-tests were employed to explore the differences. Significant variables from univariate analysis were included in binary logistic regression to model the probability of poor sleep quality. Model construction yielded odds ratios (OR) and corresponding 95% confidence intervals (95%CI) for each predictor.

## 5 Results

### 5.1 Comparison of sleep quality at the individual level in subjects with different characteristics

Participants’ Characteristicshowed the demographic information of university students in Jiangsu Province, China (**Table 1**). The result revealed that the largest proportion of participants consisted of students in their first and second years of study (90.29%). Of these participants, 69.97% were female, 61.34% were not the only child in their family, and the majority hailed from urban and suburban areas (60.39%). When it came to parental education, over half of the participants had either a high school education (68.99% and 73.98%, respectively). Furthermore, majority of the participants belonged to families with a moderate economic status (78.26%). Interestingly, only a small percentage of the participants reported smoking cigarettes (5.08%), and 31.71% of them admitted to consuming alcohol. The majority of university students experienced high levels of academic and employment-related stress (95.53% and 83.18%, respectively). It is worth noting that 56.68% of participants believed their roommates had a habit of staying up late. Additionally, over half of the participants described their physical and mental health as poor (56.63% and 50.22%, respectively). The result further revealed significant correlations between various factors and the sleep quality of university students. Specifically, the participants’ gender, grade level, hometown, father’s education level, family economic status, smoking and drinking habits, physical exercise frequency, academic and employment-related pressures, self-assessment of the dormitory environment, relationships with classmates, as well as their self-assessment of physical and mental health in comparison to others, all demonstrated associations with sleep quality.

**Table 1:** The sociodemographic characteristics of university students in this study.

Variables	N(%)	PSQI scores ≤5	PSQI scores > 5	$X^{2}$	P
Total	8458(100)	5135(60.70)	3323(39.30)		
Gender				7.914	0.005
Male	2540(30.03)	1600(62.99)	940(37.01)		
Female	5918(69.97)	3535(59.73)	2383(40.27)		
Grade				76.778	<0.001
First year	5141(60.78)	3297(64.13)	1844(35.87)		
Second year	2496(29.51)	1426(57.13)	1070(42.87)		
Third year or higher	821(9.71)	412(50.18)	409(49.82)		
Native place				8.266	0.004
Rural	3350(39.61)	2097(62.60)	1253(37.40)		
Urban	5108(60.39)	3038(59.48)	2070(40.52)		
Single-child status				22.924	<0.001
Only child	3270(38.66)	2090(63.91)	1180(36.09)		
Not only child	5188(61.34)	3045(58.69)	2143(41.31)		
Father’s education level				10.284	0.006
High school and below	5835(68.99)	3476(59.57)	2359(40.43)		
junior university	1369(16.18)	868(63.40)	501(36.60)		
Undergraduate and above	1254(14.83)	791(63.08)	463(36.92)		
Mother’s education level				5.555	0.062
High school and below	6257(73.98)	3753(59.98)	2504(40.02)		
junior university	1229(14.53)	767(62.41)	462(37.59)		
Undergraduate and above	972(11.49)	615(63.27)	357(36.73)		
Family Economy				54.099	<0.001
High	725(8.57)	492(67.86)	233(32.14)		
Middle	6619(78.26)	4066(61.43)	2553(38.57)		
Low	1114(13.17)	577(51.80)	537(48.20)		
BMI				5.717	0.057
<18.5	1586(18.75)	925(58.32)	661(41.68)		
18.5-24	5218(61.69)	3179(60.92)	2039(39.08)		
>24	1654(19.56)	1031(62.33)	623(37.67)		
Smoking				44.328	<0.001
Never	8028(94.92)	4936(61.48)	3092(38.52)		
Occasional	316(3.73)	156(49.37)	160(50.63)		
Usually	114(1.35)	43(37.72)	71(62.28)		
Drinking				48.464	<0.001
Never	5776(68.29)	3625(84.37)	2151(15.63)		
Occasional	2586(30.57)	1475(80.12)	1111(19.88)		
Usually	96(1.14)	35(63.54)	61(36.46)		
Physical exercise				49.388	<0.001
≤1 per month	1391(16.44)	728(52.34)	663(47.66)		
1-3times per week	5681(67.17)	3532(62.17)	2149(37.83)		
4-7times per week	1386(16.39)	875(63.13)	511(36.87)		
academic pressure				177.280	<0.001
Low	378(4.47)	262(69.31)	116(30.69)		
Moderate	6053(71.57)	3895(64.35)	2158(35.65)		
High	2027(23.96)	978(48.25)	1049(51.75)		
employment stress				177.819	<0.001
Low	1422(16.82)	982(69.06)	440(30.94)		
Moderate	4281(50.61)	2753(64.31)	1528(35.69)		
High	2755(32.57)	1400(50.82)	1355(49.18)		
Do you think your roommates sleep late?				127.668	<0.001
Yes	3664(43.32)	1973(53.85)	1691(46.15)		
No	4794(56.68)	3162(65.96)	1632(34.04)		
Do you think the dormitory is noisy?				172.210	<0.001
Yes	2361(27.91)	1169(49.51)	1192(50.49)		
No	6097(72.09)	3966(65.05)	2131(34.95)		
Do you think the dormitory lights are bright?				25.332	<0.001
Yes	2430(28.73)	1373(56.50)	1057(43.50)		
No	6028(71.27)	3762(62.41)	2266(37.59)		
Do you approve of the hygiene of the dormitory?				43.905	<0.001
Yes	5806(68.65)	3663(63.09)	2143(36.91)		
No	2652(31.35)	1472(55.51)	1180(44.49)		
Schoolmate relationship				147.140	<0.001
Good	5877(69.48)	3810(64.83)	2067(35.17)		
General	2454(29.02)	1277(52.04)	1177(47.96)		
Poor	127(1.50)	48(37.80)	79(62.20)		
Self-assessed health status				349.091	<0.001
Good	3668(43.37)	2604(70.99)	1064(29.01)		
General	4396(51.97)	2396(54.50)	2000(45.50)		
Poor	394(4.66)	135(34.26)	259(65.74)		
Self-assessed mental health status				565.378	<0.001
Good	4210(49.78)	3063(72.76)	1147(27.24)		
General	3787(44.77)	1921(50.73)	1866(49.27)		
Poor	461(5.45)	151(32.75)	310(67.25)		

### 5.2 Psychological resilience and mobile phone addiction with sleep quality

The total and domain scores from the MPAI and CD-RISC assessments between individuals with and without sleep disorders were showed in **Table 2**. The mean total score on the MPAI for individuals with sleep disorders was 47.89 ± 13.87, while the mean score for those without sleep disorders was 42.70 ± 13.24. Similarly, for the CD-RISC total score, individuals with sleep disorders obtained a mean score of 57.07 ± 17.17, whereas those without sleep disorders had a mean score of 63.22 ± 18.53. Across all four domains of the MPAI, patients with sleep disorders exhibited significantly higher scores compared to patients without sleep disorders (*P<* 0.001). Conversely, patients with sleep disorders demonstrated significantly lower scores across all three domains of the CD-RISC compared to patients without sleep disorders (*P<* 0.001).

**Table 2:** Psychological resilience score/Mobile phone addiction score for participants with sleep quality (n=8258).

Variables	PSQI scores ≤ 5 ( $\bar{x}$ ± SD)	PSQI scores > 5 ( $\bar{x}$ ± SD)	*t*	*P*
MPAI				
Total	42.70 ± 13.24	47.89 ± 13.87	-17.545	<0.001
Inability to Control Craving	16.03 ± 5.25	18.04 ± 5.52	-16.790	<0.001
Withdrawal and Escape	9.53 ± 4.17	10.92 ± 4.27	-14.845	<0.001
Anxiety and Feeling Lost	8.58 ± 3.21	9.46 ± 3.12	-12.452	<0.001
Productivity Loss	8.56 ± 3.05	9.48 ± 2.96	-13.825	<0.001
CD-RISC				
Total	63.22 ± 18.53	57.07 ± 17.17	15.339	<0.001
Optimism	9.66 ± 3.13	8.81 ± 3.04	12.401	<0.001
Self-improvement	21.45 ± 6.15	19.40 ± 5.71	15.615	<0.001
Tenacity	31.0(26.0,39.0)	26.0(25.0,34.0)	15.007	<0.001

### 5.3 Logistic regression of influential factors contributing to sleep quality

The risk and protective factors that influence the quality of sleep among university students were showed in **Table 3**. Female students have a higher likelihood of experiencing poor sleep quality compared to male students (OR = 1.238; 95% CI, 1.109-1.382). Seniors are 1.6 times more than juniors to likely to have sleep disorders (OR = 1.582; 95% CI, 1.344-1.863). Non-only children are more susceptible to poor sleep quality (OR = 1.195; 95% CI, 1.077-1.326). Regular smoking increases the risk of sleep disorders (OR = 1.833; 95% CI, 1.181-2.847). Similarly, university students who consume alcohol regularly are 1.74 times more likely to develop sleep disorders than those who abstain from alcohol (OR = 1.737; 95% CI, 1.065-2.833). Higher levels of physical activity are associated with a reduced risk of sleep disorders, as university students who engage in frequent exercise are less likely to experience sleep disturbances compared to those with less physical activity (OR = 0.921; 95% CI, 0.779-1.090). Additionally, university students facing high academic stress are 1.2 times more likely to experience sleep disturbances than those with lower stress levels (OR = 1.326; 95% CI, 1.012-1.736). Similarly, elevated employment stress is associated with a 1.4-fold increase in the likelihood of sleep disturbances (OR = 1.352; 95% CI, 1.156-1.582). Students whose roommates have earlier bedtimes are less likely to experience sleep disturbances (OR = 0.799; 95% CI, 0.718-0.888), and those residing in quieter dormitories have a lower likelihood of acquiring sleep disorders (OR = 0.732; 95% CI, 0.647-0.828). Dissatisfaction with dormitory hygiene increases the likelihood of poor sleep quality by 1.1 times compared to being satisfied with dormitory hygiene (OR = 1.140; 95% CI, 1.028-1.265). Students with poor self-rated physical health face a twofold higher risk of sleep disorders than those with good self-rated physical health (OR = 1.969; 95% CI, 1.533-2.529). Similarly, students with poor self-rated mental health are 2.9 times more likely to have a sleep disorder compared to those with good self-rated mental health (OR = 2.924; 95% CI, 2.309-3.702). A high score on the MPAI (OR = 1.029; 95% CI: 1.026-1.033) was associated with a slightly increased risk of poor sleep quality, suggesting that individuals with higher MPAI values may be more likely to experience sleep disturbances. On the other hand, a higher score on the CD-RISC (OR = 0.982; 95% CI: 0.979-0.984) was linked to a small but statistically significant decrease in the odds of poor sleep quality, indicating that greater resilience, as measured by the CD-RISC, may serve as a protective factor against sleep quality issues.

**Table 3:** Results of logistic regression analysis of sleep quality associated with demographic, psychological and behavioral factors.

Variables		Reference	β	P	OR (95% CI)
gender	Female	Male	0.214	<0.001	1.238 (1.109-1.382)
Grade	Second year	First year	0.205	<0.001	1.228 (1.105-1.364)
	Third year or higher		0.459	<0.001	1.582 (1.344-1.863)
Native place	Urban	Rural	0.052	0.353	1.054 (0.943-1.177)
Single-child status	Not only child	Only child	0.178	0.001	1.195 (1.077-1.326)
Father’s education level	junior university	High school and below	-0.060	0.397	0.942 (0.821-1.081)
	Undergraduate and above		-0.035	0.644	0.965 (0.830-1.122)
Family Economy	Middle	High	0.028	0.764	1.028 (0.856-1.235)
	Low		0.098	0.393	1.103 (0.881-1.381)
Smoking	Occasional	Never	0.265	0.038	1.304 (1.015-1.676)
	Usually		0.606	0.007	1.833 (1.181-2.847)
Drinking	Occasional	Never	0.178	0.001	1.195 (1.075-1.329)
	Usually		0.552	0.027	1.737 (1.065-2.833)
Physical exercise	1-3times per week	≤1 per month	-0.135	0.042	0.873 (0.767-0.995)
	4-7times per week		-0.082	0.338	0.921 (0.779-1.090)
academic pressure	Moderate	Low	0.016	0.900	1.016 (0.789-1.309)
	High		0.282	0.040	1.326 (1.012-1.736)
employment stress	Moderate	Low	0.089	0.228	1.093 (0.946-1.262)
	High		0.302	<0.001	1.352 (1.156-1.582)
Do you think your roommates sleep late?	No	Yes	-0.225	<0.001	0.799 (0.718-0.888)
Do you think the dormitory is noisy?	No	Yes	-0.312	<0.001	0.732 (0.647-0.828)
Do you think the dormitory lights are bright?	No	Yes	-0.061	0.270	0.940 (0.843-1.049)
Do you approve of the hygiene of the dormitory?	No	Yes	0.131	0.013	1.140 (1.028-1.265)
schoolmate relationship	General	Good	0.099	0.071	1.104 (0.991-1.230)
	Poor		0.073	0.727	1.075 (0.716-1.616)
Self-assessed health status	General	Good	0.201	0.001	1.222 (1.088-1.393)
	Poor		0.678	<0.001	1.969 (1.533-2.529)
Self-assessed mental health status	General	Good	0.669	<0.001	1.951 (1.738-2.191)
	Poor		1.073	<0.001	2.924 (2.309-3.702)
MPAI			0.029	<0.001	1.029 (1.026-1.033)
CD-RISC			-0.019	<0.001	0.982 (0.979-0.984)

## 6 Discussion

In a cross-sectional survey of 8,458 university students in Jiangsu Province, China, we found that sleep disorders were common among university students. Physical activity and high psychological resilience had a significant positive and protective effect on sleep quality. Higher grades, not being an only child, academic and employment stress, smoking and drinking habits, roommates staying up late, noisy dormitory environment, poor dormitory hygiene, self-rated physical and mental health problems and high MPAI scores had significant negative effects on sleep quality.

This study revealed that 39.30% of the participants experienced poor sleep quality as determined by the PSQI scale with a critical sleep disorders value of 5. This finding notably contrasts with research conducted in other regions of China. For instance, a survey conducted in Shanghai in 2022 during the COVID-19 period reported a prevalence of sleep disorders of 90.2% among dialysis patients (40), and a study conducted in Beijing in 2021 identified a prevalence of 53.2% (41). Meanwhile, a survey conducted in Changsha reported that only 9.8% of university students suffer from sleep disorders (42), suggesting a substantial variation. Furthermore, the prevalence of this health problem is 52.4% in Turkey (43). Such disparate findings could be ascribed to the multitude of factors influencing sleep quality, including but not limited to, the demographic and health status of the surveyed population, the period during which the data was collected, and distinctive regional or cultural influences. Another important consideration is the different psychometric thresholds applied to the PSQI or other sleep assessment tools, as which can influence the classification of sleep quality and the reported prevalence of disorders.

In our study, we identified several demographic variables that were significantly linked to the prevalence of sleep disorders. Specifically, our analysis supports existing literature (44) which is showing that female students are more susceptible to experiencing sleep disturbances, a trend that has been consistently observed across various studies. One study found that the higher prevalence of sleep disorders among female students may be associated with premenstrual syndrome (PMS) (45). It is noteworthy that our sample had an overrepresentation of females, which may skew the results. This overrepresentation could limit the generalizability of our findings to all students. Further research with a more balanced gender sample is necessary. Furthermore, upperclassmen exhibited a significantly higher prevalence of sleep disorders compared to their junior peers. This could be attributed to the escalating pressure associated with graduation, which typically brings about more intense academic workloads and career-related stress. Family structure also appeared to influence sleep quality, as students who were not the only child in their family tended to report more sleep problems. This might be related to the dynamics of parental attention and emotional support, wherein non-only children may receive less focused support, leading to enhanced anxiety which can negatively affect sleep. Lifestyle factors such as smoking and alcohol consumption were found to correlate significantly with poor sleep quality, corresponding to previous research (18). In stark contrast, a beneficial link was noted between physical activity and sleep quality. Regular participation in physical activities was inversely related to poor sleep quality, suggesting that a more active lifestyle could act as a protective factor against sleep disorders, corroborating the findings from other studies (26).

University students facing higher levels of academic and employment stress were more prone to experiencing poor sleep quality. Academic pressures coupled with job-related concerns can lead to heightened worries about the future. Such stress generates considerable psychological strain, which can directly interfere with the ability to obtain restful sleep. The anticipation of future events and obligations often keeps the mind actively engaged at night, preventing the relaxation necessary to initiate sleep.

Environmental elements in a university setting can also be disruptive to sleep. The sleeping habits of roommates, noisy dormitory environments, and poor dormitory hygiene can hinder university students from attaining a conducive sleeping environment, resulting in compromised sleep quality.

Lastly, students who rated their physical and mental health poorly also exhibited noticeable sleep disorder problems. These students may encounter mental health issues such as stress, anxiety, and depression, which can disrupt their ability to fall asleep or maintain good sleep quality. Additionally, physical health problems like chronic pain, digestive issues, and respiratory difficulties can adversely affect sleep patterns.

Furthermore, our study revealed that the level of mobile phone addiction and psychological resilience had significant correlations with sleep quality among university students. Specifically, we observed that university students with higher levels of mobile phone addiction were more prone to experiencing poor sleep quality. Several studies have indicated that the blue light emitted by smartphones is associated with sleep quality (46). When the body is exposed to blue light at night, it disrupts the body’s biological clock. This is because that blue light from smartphones suppresses the brain’s production of melatonin, which in turn affects our ability to fall asleep (47). Conversely, psychological resilience, which serves as a beneficial psychological asset, had a protective influence on sleep quality. University students with higher psychological resilience demonstrated a decreased likelihood of experiencing poor sleep quality. These findings were further supported by a related study (48).

Enhancing the sleep quality of university students is essential and effective strategies must be deployed. One key initiative is the provision of sleep education (49), which equips students with knowledge regarding the critical role of sleep for both brain function and overall well-being. Practical sleep tips can also be provided to assist students in establishing healthy sleep habits. Additionally, advocating for a harmonious lifestyle that includes routine physical activity and balanced nutrition is instrumental in synchronizing the body’s circadian rhythm, thus facilitating easier transitions into slumber and refining overall sleep quality. The optimization of the sleep environment is eqully critical (50). Schools can create quiet study and living spaces, while employing resources such as blackout curtains, earplugs, and noise machines to foster a tranquil and comfortable sleeping environment. Furthermore, It was found that psychological resilience had a closed association with sleep quality, and enhancing psychological resilience could mitigate the direct and indirect impacts of stressful life events on sleep quality (35). It is vital to provide psychological support and equip students with stress management techniques to mitigate the impact of stress on sleep quality. By offering psychological counseling and support services, schools can aid students in effectively coping with stress and anxiety. To combat the pervasive issue of mobile phone addiction, it is prudent for both educational institutions and households to formulate and enforce sensible mobile phone usage policies. Encouraging students to abstain from mobile phone usage one hour before bedtime and employing mobile phone applications to monitor usage time prove to be beneficial. Presenting an assortment of captivating alternatives such as athletic competitions and social get-togethers serves to shift students’ focus away from their digital screens, thus nurturing healthier lifestyle choices. In parallel, the establishment of set digital downtime encourages students to temporarily detach from their electronic gadgets, granting them the opportunity to relax and enhance their sleep quality.

## 7 Conclusions

The overall prevalence of poor sleep quality was 39.30%, with a detection rate of 40.27% among female university students and 37.01% among male university students. Binary logistic regression analysis revealed that physical activity and high psychological flexibility significantly improved sleep quality, providing a protective effect. Additionally, higher grade level, not being an only child, academic and employment stress, smoking and drinking habits, roommates staying up late, a noisy dormitory environment, poor dormitory hygiene, self-rated physical and mental health issues, and a high MPAI score were identified as risk factors associated with sleep quality. It is imperative to take actionable measures to improve the sleep quality of university students in Jiangsu Province, China.

### 7.1 Limitations

Several limitations should be noted in our study. Firstly, the study’s cross-sectional design limits our ability to establish causality. Longitudinal research in the future would be beneficial in determining the predictors of sleep quality rather than merely assessing correlations. Secondly, the reliance on self-reported questionnaires for data collection might have introduced recall bias. Future studies could integrate objective methods such as sleep monitoring devices and activity trackers to obtain more accurate sleep data. Thirdly, we acknowledge that the gender ratio in our study was skewed, with an overrepresentation of female participants. This imbalance could introduce a selection bias affecting the reported prevalences of sleep issues, which may not accurately reflect the broader population. We recommend that subsequent research efforts aim for a balanced gender mix to diminish potential gender-based bias and enhance the representativeness of the findings. Another limitation is that we were unable to track the exact number of students who accessed the survey link in the open-ended online survey, and therefore could not calculate a traditional response rate.

## Data availability statement

The raw data supporting the conclusions of this article will be made available by the authors, without undue reservation.

## Ethics statement

The studies involving humans were approved by Ethics Committee of Xuzhou Medical University. The studies were conducted in accordance with the local legislation and institutional requirements. Written informed consent for participation in this study was provided by the participants’ legal guardians/next of kin.

## Author contributions

BH: Conceptualization, Writing – review & editing. QW: Data curation, Investigation, Software, Writing – original draft. WY: Data curation, Writing – review & editing. HZ: Data curation, Writing – review & editing. DY: Conceptualization, Funding acquisition, Writing – review & editing.
